# Supplementary material for: Synchronous Head and Neck Cancer and Superficial Esophageal Squamous Cell Neoplasm: Endoscopic Treatment or No Treatment for the Superficial Esophageal Neoplasm
Source: Cancers (Basel). 2023 Feb 8;15(4):1079. doi: 10.3390/cancers15041079 (PMC9954443; doi:10.3390/cancers15041079)
Supplement: Supplementary file 1 [file cancers-15-01079-s001.zip › cancers-2179811-supplementary.pdf]

Figure S1. Therapeutic guideline for head and neck cancer in our institution

Squamous Cell Carcinoma of Head and Neck (SCCHN)	Resectable, Oral Cavity	→ Surgery	No following risk factor	→ Observation	p.3
			Tongue cancer with extrinsic muscle extension; pT4 (exclude tumor>4cm and DOI>10mm), or pT3N1, or pT1-2N1 (level Ⅳ/Ⅴ), or close margin ≤ 2mm, or poorly differentiation with tumor depth ≧ 4mm, or =2 minor risk factors	→ Adjuvant RT	
			ENE (+), or margin (+), or ≧pN2b, or ≧3 minor risk factors	→ Adjuvant CCRT	
	Unresectable/Inoperable/ Low surgical curability, Oral Cavity	→ CCRT<br>→ Bio -radiotherapy<br>→ Neoadjuvant CT → CCRT or Bio -radiotherapy			p.4-5
	Resectable, Oropharynx/Hypopharynx/Larynx	→ Surgery	No following risk factor	→ Observation	p.6
			pT4, or pT3N1, pT1-2N1 (level Ⅳ/Ⅴ) or close margin ≤ 2mm, or poorly differentiation, or = 2 minor risk factors	→ Adjuvant RT	
			ENE (+), or margin (+), or pN2b, or ≧3 minor risk factors	→ Adjuvant CCRT	
	Resectable, Oropharynx /Hypopharynx/Larynx	→ Endoscopic surgery or RT alone<br>→ CCRT<br>→ Bio -radiotherapy			p.7-10
	Unresectable/Inoperable/Low surgical curability, Oropharynx/Hypopharynx/Larynx	→ CCRT<br>→ Bio -radiotherapy<br>→ Neoadjuvant CT → CCRT or Bio -radiotherapy			p.11-12
	Recurrent/metastatic (if not considered with surgery or radiotherapy)	→ CT, Molecular Targeted therapy, Immuno-Oncologic therapy<br>→ Clinical trials			p.13
